# Curve progression after long-term brace treatment in adolescent idiopathic scoliosis: comparative results between over and under 30 Cobb degrees - SOSORT 2017 award winner

**DOI:** 10.1186/s13013-017-0142-y

**Published:** 2017-10-30

**Authors:** Angelo G. Aulisa, Vincenzo Guzzanti, Francesco Falciglia, Marco Galli, Paolo Pizzetti, Lorenzo Aulisa

**Affiliations:** 1 U.O.C. of Orthopedics and Traumatology, Children’s Hospital Bambino Gesù, Institute of Scientific Research, P.zza S. Onofrio 4, 00165 Rome, Italy; 2 University of Cassino, 03043 Cassino, FR Italy; 3 Department of Orthopedics, University Hospital “Agostino Gemelli”, Catholic University of the Sacred Heart School of Medicine, 00168 Rome, Italy

**Keywords:** Brace treatment, Adolescent idiopathic scoliosis, P.A.S.B, Long term, Follow-up

## Abstract

**Background:**

The factors influencing curve behavior following bracing are incompletely understood and there is no agreement if scoliotic curves stop progressing with skeletal maturity. The aim of this study was to evaluate the loss of the scoliotic curve correction in patients treated with bracing during adolescence and to compare patient outcomes of under and over 30 Cobb degrees, 10 years after brace removal.

**Methods:**

We reviewed 93 (87 female) of 200 and nine patients with adolescent idiopathic scoliosis (AIS) who were treated with the Lyon or PASB brace at a mean of 15 years (range 10–35). All patients answered a simple questionnaire (including work status, pregnancy, and pain) and underwent clinical and radiological examination. The population was divided into two groups based on Cobb degrees (< 30° and > 30°). Statistical analysis was performed to test the efficacy of our hypothesis.

**Results:**

The patients underwent a long-term follow-up at a mean age of 184.1 months (±72.60) after brace removal. The pre-brace scoliotic mean curve was 32.28° (± 9.4°); after treatment, the mean was 19.35° and increased to a minimum of 22.12° in the 10 years following brace removal. However, there was no significant difference in the mean Cobb angle between the end of weaning and long term follow-up period (*p* = 0.105). The curve angle of patients who were treated with a brace from the beginning was reduced by 13° during the treatment, but the curve size lost 3° at the follow-up period.

The groups over 30° showed a pre-brace scoliotic mean curve of 41.15°; at the end of weaning, the mean curve angle was 25.85° and increased to a mean of 29.73° at follow-up; instead, the groups measuring ≤ 30° showed a pre-brace scoliotic mean curve of 25.58°; at the end of weaning, it was reduced to a mean of 14.24° and it increased to 16.38° at follow-up.

There was no significant difference in the mean progression of curve magnitude between the ≤ 30° and > 30° groups at the long-term follow-up.

**Conclusions:**

Scoliotic curves did not deteriorate beyond their original curve size after bracing in both groups at the 15-year follow-ups. These results are in contrast with the history of this pathology that normally shows a progressive and lowly increment of the curve at skeletal maturity. Bracing is an effective treatment method characterized by positive long-term outcomes, including for patients demonstrating moderate curves.

## Background

Adolescent idiopathic scoliosis (AIS) is a three-dimensional spinal deformity that it is characterized by lateral curvature of the spine and vertebral rotation. The severity of AIS varies greatly, and not all the curves have a progression that requires treatment [1, 2].

The most common and conservative approach to treatment of AIS is using a brace to prevent the progression of curvature and in select cases, to obtain a partial recovery of the curve [3–7]. The efficacy of bracing is correlated with longer daily application time and to patient adherence to treatment plans [8–10]. Literature shows the factors that influence curve behavior following bracing are not fully understood, but they are crucial to the prognosis of patients with AIS [10, 11]. Moreover, there is no agreement if scoliotic curves stop progressing after bracing at skeletal maturity.

The aims of this study were:

- To evaluate the loss of the scoliotic curve correction at long term follow-up in a cohort of patients treated with bracing during adolescence;
- To compare the outcomes of sub-group patients: (1) Over and under 30 Cobb degrees at start of treatment, to determine whether the initial curve’s gravity could influence long-term results; and (2) over and under 30 Cobb degrees at end of weaning.

## Methods

### Patient population

This is a retrospective study based on an ongoing database including 1512 patients treated for idiopathic scoliosis between 1980 and 2016. Informed consent was obtained by all participants. This study was conducted in accordance with the World Medical Association Declaration of Helsinki of 1975, as revised in 1983, and all the participants signed an informed consent to allow the use of clinical data for research purposes.

A total of 209 scoliotic patients treated with the progressive action short brace (PASB) or Lyon brace were contacted a minimum of 10 years from the end of treatment (range 10–35). Ninety-three patients (87 female) responded to the long-term follow-up examination.

All patients presented at the beginning of treatment, AIS, with curves ranging in magnitude between 20° and 55° Cobb. Age at the beginning of treatment was 10–14 years, with Risser scores between 0 and 2.

### Bracing protocol

All patients were prescribed a full-time (i.e., maximum 22 h daily, minimum 18 h daily) brace. For the study, patients showing curve angles < 25°, progression was assessed using two consecutive radiographs taken at 6-month intervals. Progression was defined as an increase greater than 5° in both curve magnitude (Cobb’s method) and apical torsion (Perdriolle’s method) in an immature skeleton. Weaning was started when ring-apophysis fusion [12] was seen on a laterolateral view radiograph and consisted of 2 to 4 h of bracing reduction at 4-month intervals. Short term follow-up was discontinued 5 years after brace removal. Radiology reports with measurements of the deformity were available for all patients.

### Follow up after 10 years since brace removing

Ninety-three patients were evaluated at long-term follow-up. Demographic characteristics were obtained. Patients were observed in the standing erect position and during the forward bending test.

Full-length anteroposterior (AP) and lateral view standing radiographs were taken. The AP view was used to obtain the curve magnitude (CM, Cobb’s method) and torsion of the apical vertebra (TA, Perdriolle’s method) [13, 14]. Measurements were obtained by two independent observers (two senior surgeons) and the end vertebrae were preselected to reduce interobserver bias [14].

All patients answered a simple questionnaire:

- Work Status (yes or no, full or part-time)
- Pregnancy (yes or no, born children)
- Back Pain (yes or no)

### Sub group analysis

The patients were divided in sub-groups based on Cobb degrees. Those with curves ≤ 30 Cobb degrees and those with curves > 30 Cobb degrees at the beginning of treatment and at end of treatment.

### Statistical analysis

Standard statistical methods have been used for descriptive statistics. Normally distributed continuous variables were analyzed by using an independent sample *t* test. Changes in CM and TA from beginning to follow-up were assessed via one-way analysis of variance for repeated measures. Mean differences between time points and 95% confidence intervals were calculated. Correlations between changes in CM at the start of bracing, at the end of weaning, and at follow-up were determined via the Pearson test. All analyses were performed according to the intention-to-treat principle. All tests were two-sided, with significance set at a *P* value less than 0.05. Results are presented as mean ± standard deviation (SD).

## Results

### Demographics

Ninety-three patients (females: 87) with a mean age 32.58 (±5.2) years were studied. The mean pre-brace scoliotic curve was 32.28 (± 9.4) degrees, and the mean Perdriolle score of the apical vertebra of the scoliotic deformity was 13.86 (± 5.04).

Twenty-five patients had a thoracic curve, 40 a thoracolumbar or lumbar curve, and 28 had a double primary curve.

Fifty-three patients (57%) had pre-brace Cobb angle less than 30°, and 40 (43%) had a Cobb angle greater than 30°.

Mean time of brace application was 5.28 (±2.23) years. Patients underwent long-term follow-up at a mean of 15.33 (±5.22) years after brace removal.

### Cohort results

In all 93 patients, the mean scoliotic curve was reduced of 13.41° (±8.1) at the end of weaning.

**Fig. 1:**
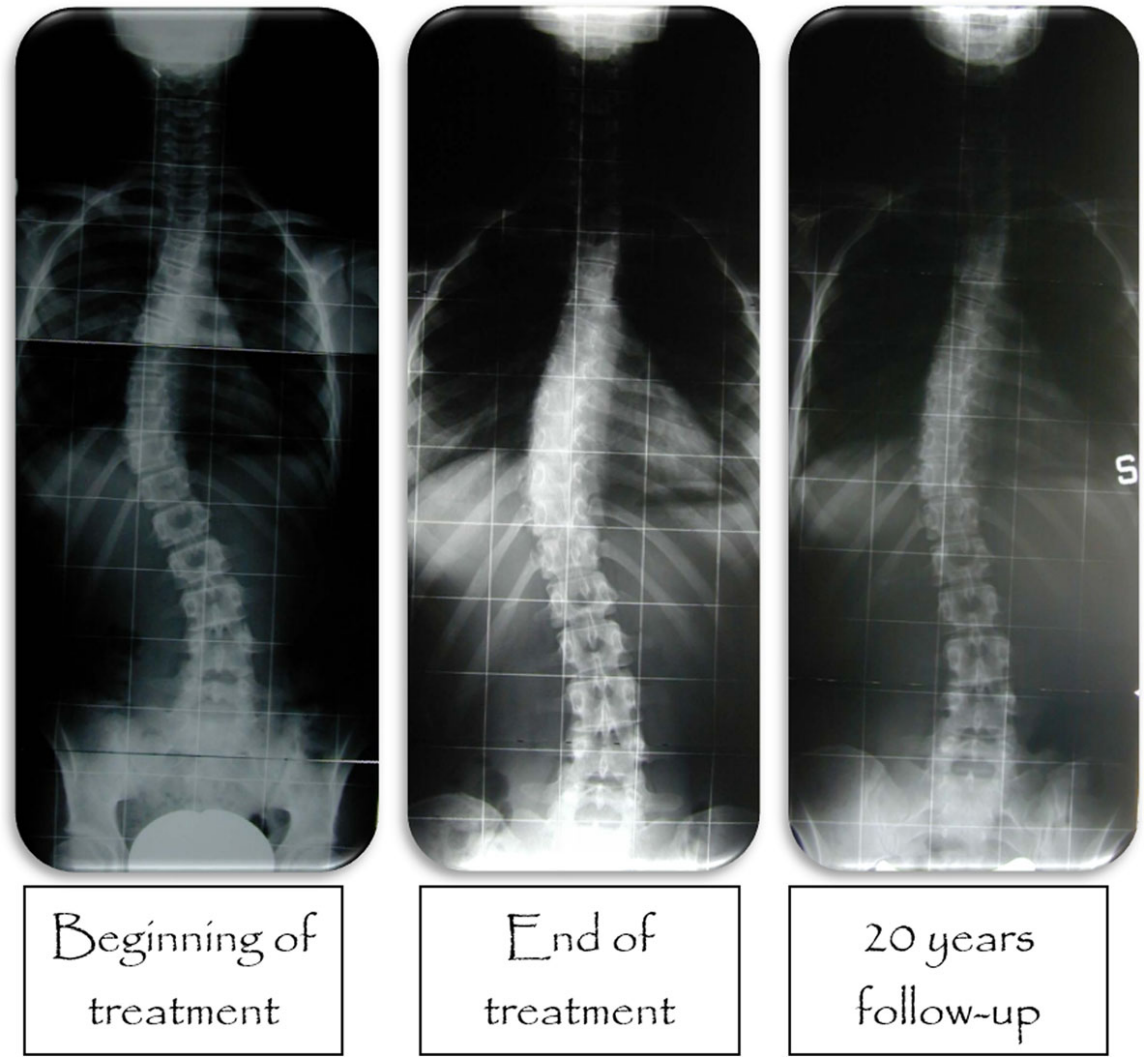
Typical radiological trend in Cobb degrees of all samples

The mean pre brace Cobb angle of 32.17° (±9.12) was reduced to 19.39° (±10.8) following brace removal, it was 20.67° (±11.2) at the time of the short term follow-up (5 years) and increased to 22.12° (±12.11) at long-term follow-up. However, there was no significant difference in the mean Cobb angle between the end of weaning and long term follow up period (*p* = 0.105) (Fig. 1).

Seventy-three patients (78.5%) completed the long term follow up with less than 30°Cobb angle, 11 (11.8%) between 30 and 40° and nine (9.7%) with a Cobb angle greater than 40°. No patients at the long term follow-up demonstrated curve angles greater than 50°.

### Comparison of ≤ 30° vs > 30° at begging of treatment

Fifty-three patients (57%) had curve with Cobb degrees ≤ 30° whereas 40 patients had a curve (43%) > 30°.

**Fig. 2:**
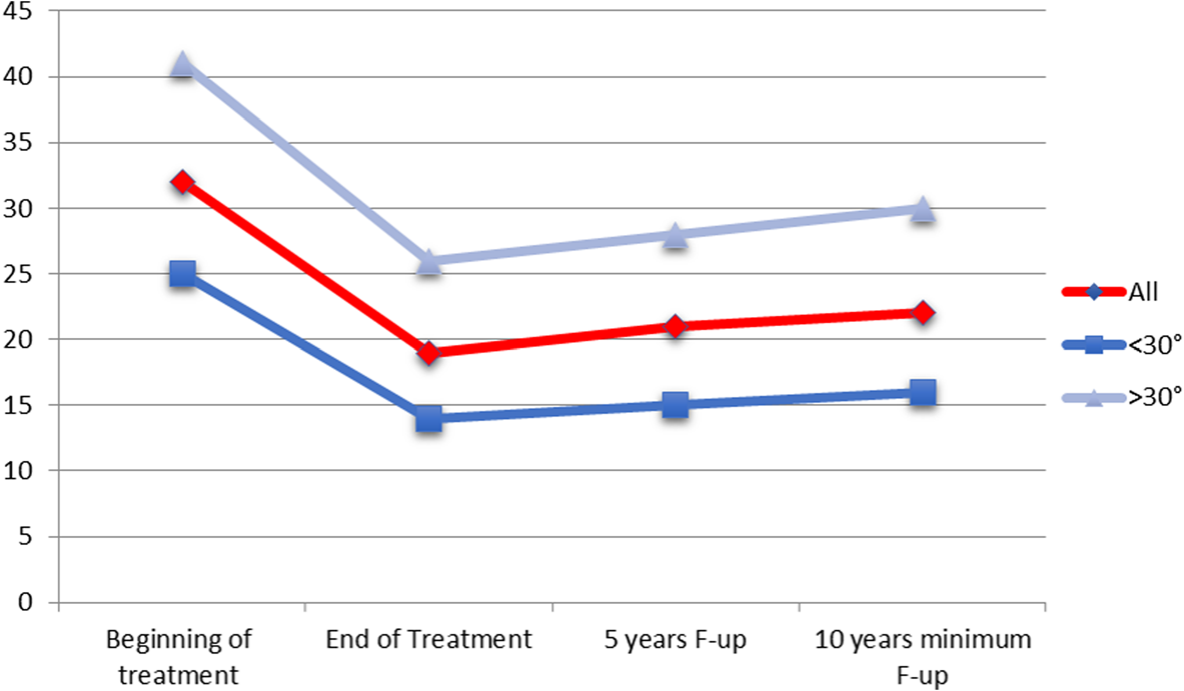
Radiological trend in Cobb degrees of the all sample and of two groups < 30° and > 30°

The group greater than 30° showed a pre-brace scoliotic mean curve of 41.15°; at the end of weaning, it was reduced to 25.85° and it increased to 29.73° at long-term follow-up; instead, the group < 30° showed a pre-brace scoliotic mean curve of 25.58°; at the end of weaning, it was reduced to 14.24° and it increased to 16.38° at long term follow-up (Table 1 and Fig. 2).

**Table 1:** Demographic and radiological characteristics of the study sample

	Beginning of treatment (*t*_1_)	End of treatment (*t*_2_)	5 years follow-up (*t*_3_)	10 years minimum follow-up (*t*_4_)
Age (years)	11.1 ± 2.4	17.1 ± 2.0	22.1 ± 2.5	32.4 ± 5.1
Cobb degrees	32.28 ± 9.4	19.4 ± 10.8	20.7 ± 11.2	22.1 ± 12.1
Perdriolle degrees	13.9 ± 5.0	9.9 ± 6.2	10,1 ± 6.9	10.4 ± 6.2

Significant differences were determined for CM across Cobb at beginning and at the end of weaning. Instead, insignificant differences were determined for CM between the end of weaning and the long-term follow-up period (Tables 2, 3).

**Table 2:** Differences in C_M_ across *t*_1_–*t*_4_ in < 30° as determined by one-way ANOVA with Bonferroni’s post test

Cobb < 30°	Mean Diff,	t	*P* < 0.05	Summary	95% CI of diff
Beginning of treatment (*t*1) vs end of treatment (*t*2)	11.3	6.90	Yes	***	6.96 to 15.7
Beginning of treatment (*t*1) vs 5 years follow-up (*t*3)	10.5	6.49	Yes	***	6.16 to 14.7
Beginning of treatment (*t*1) vs 10 years minimum follow-up (*t*4)	9.21	5.72	Yes	***	4.92 to 13.5
End of treatment (*t*2) vs 5 years follow-up (*t*3)	−0,887	0.540	No	ns	−5.27 to 3.49
End of treatment (*t*2) vs 10 years minimum follow-up (*t*4)	−2.13	1.30	No	ns	−6.51 to 2.25
5 years follow-up (*t*3) vs 10 years minimum follow-up (*t*4)	−1.25	0.773	No	ns	−5.54 to 3.05

***significant

^ns^not significant

**Table 3:** Differences in C_M_ across *t*_1_–*t*_4_ in > 30° as determined by one-way ANOVA with Bonferroni’s post test

Cobb >30°	Mean Diff,	t	*P* < 0.05	Summary	95% CI of diff
Beginning of treatment (*t*1) vs End of treatment (*t*2)	15.3	7.36	Yes	***	9.75 to 20.9
Beginning of treatment (*t*1) vs 5 years follow-up (*t*3)	13.2	6.37	Yes	***	7.63 to 18.7
Beginning of treatment (*t*1) vs 10 years minimum follow-up (*t*4)	11.4	5.53	Yes	***	5.91 to 16.9
End of treatment (*t*2) vs 5 years follow-up (*t*3)	−2.15	1.04	No	ns	−7.71 to 3.40
End of treatment (*t*2) vs 10 years minimum follow-up (*t*4)	−3.88	1.87	No	ns	−9.43 to 1.67
5 years follow-up (*t*3) vs 10 years minimum follow-up (*t*4)	−1.73	0.835	No	ns	−7.24 to 3.79

***significant

^ns^not significant

The mean curve correction was 10.94° in the group with Cobb angles ≤ 30° and was 15.3° in the group with Cobb angles > 30°. There was no statistically significant difference in the mean curve correction between the two groups at short term follow-up (Fig. 1).

Long-term follow up revealed a moderate increase in the Cobb angle in both groups. The mean Cobb angle increase was 2.14° in the group with Cobb angles ≤ 30° and 3.88° in the group with Cobb angles > 30°. The difference between groups was not statistically significant (*p* = 0.87).

### Comparison of ≤ 30° vs > 30° at end of treatment

Seventy five patients (81%) had curve angle ≤ 30°, whereas 18 patients (19%) had a curve angle > 30° at the end of conservative treatment.

The group greater than 30° showed a pre-brace scoliotic mean curve of 43.94°, at the end of weaning it was reduced to 34.89° and it increased to 38.39° at long term follow-up; instead the group < 30° showed a pre-brace scoliotic mean curve of 29.35°, at the end of weaning it was reduced to 15.05° and it increased to 18.21° at long-term follow-up. No significant differences were determined for CM between end of weaning and long-term follow up period.

Long term follow-up revealed a moderate increase in the Cobb angle in both groups. The mean Cobb angle increase was 3.16° in the group with Cobb angles ≤ 30° and 3.50° in the group with Cobb angles > 30°. Difference between groups was not statistically significant.

### Demographics

Sixty-one patients were employed full-time, 19 were employed part-time, and 13 were unemployed. Twenty-one patients experienced pregnancy. Pain, related to instability of the spine, was present in 12 patients (3 cases were described as chronic).

## Discussion

The main objective of this study was to evaluate the loss of correction at long term follow-up and to analyze our case series.

Past studies of AIS showed a progression of the curve also at the end of the growth, but the degree of progression was not clear. Weinstein reported that “even in progressive curves it cannot be predicted, for example, whether a progressive 30° curve’s natural history would be to progress to 38° or to 78°” [15]. Instead, Bjerkreim [16] reported in his paper about the Progression in Untreated Idiopathic Scoliosis that curve progression was 3° per year before 20 of age and 1° per year after 20 years. Curves less than 40 degrees increased significantly less than larger curves and curves measuring from 60° to 80° increased the most.

Unfortunately, there are a limited number of studies that support the corrective effect of bracing at the time of the long-term follow-up compared to the natural progression of untreated curves [17–21].

Pellios et al. [17] reported the long term results of 77 patients suffering from AIS at 25 years after Boston brace removal. The initial Cobb angle of 28.2° (±8.7) was reduced during brace application to 17.3° (±9.2) then increased at the 25 year follow-up to 25.4° (±13.8). The mean loss of correction at 25 years after brace discontinuation was 8.1°. Nachemson [20] confirmed similar long-term results of 109 patients, the mean loss of correction at 22 years after brace discontinuation was 7.9°. Lange et al. [21] showed better results in a similar study of 215 patients at 25 years after Boston brace removal. They reported the mean Cobb angle deterioration at the long term evaluation was 4.1° after brace removal.

In our study, the mean pre-brace Cobb angle of 32.17° (±9.12) was reduced during brace application to 19.39° (±10.8). It increased slightly at the short time follow-up to 20.67° (±11.2) and further increased at the 15-year follow-up to 22.12° (±12.11). However, the mean pre-brace Cobb angle was not significantly different at the 15-year follow-up, demonstrating stability with a loss of correction of 2.7°.

78.5% of our cohort completed the long-term follow up with less than 30° Cobb angle. Therefore, our results are slightly better than those published in the literature regarding the course of curve progression following brace removal. Bracing seems to be an effective treatment method, with good long-term results also shown in moderate curves.

Furthermore, the results from the subgroups at long-term follow-up revealed a slight increase in the Cobb angle in both groups. The increase was not significantly different at 15 years follow-up and the difference between groups, 2.14° (Cobb ≤ 30°) versus 3.88° (Cobb > 30°), was not statistically significant. These results are in contrast with past studies that showed a progressive increment of the curve at skeletal maturity in those that were classified as moderate [1, 22]. Reasons effecting the stability of the curve after the brace treatment were not clear from the results but may be related to stiffness of the treated curves.

Using collected demographic data, we found no significant difference in both pregnancy and pain between groups.

A limitation of our study was that the cohort is still young with a mean of 32 years. It would be useful to study patients older than 50 years, in which the degenerative processes of the spine are more evident and important. Even more interesting would be studying the behavior thoracolumbar curves; in fact, the three cases of chronic pain related to instability, due to rotational subluxation, were thoracolumbar curves.

## Conclusions

The results demonstrate slight loss of correction 15 years post bracing. We found no difference in terms of long-term results and progression between patients with ≤ 30° vs > 30° Cobb angles. In conclusion, bracing could be effective for long-term in patients with adolescent idiopathic scoliosis.

## Abbreviations

AIS: Adolescent idiopathic scoliosis
AP: Anterior-posterior
CM: Cobb’s method
LL: Latero-lateral
PASB: Progressive action short brace
SOSORT: Society on Scoliosis Orthopedic and Rehabilitation Treatment
SRS: Scoliosis Research Society
TA: Perdriolle’s method
